# Directed Information Flow Analysis Reveals Muscle Fatigue-Related Changes in Muscle Networks and Corticomuscular Coupling

**DOI:** 10.3389/fnins.2021.750936

**Published:** 2021-09-09

**Authors:** Tie Liang, Qingyu Zhang, Lei Hong, Xiaoguang Liu, Bin Dong, Hongrui Wang, Xiuling Liu

**Affiliations:** ^1^Institute of Electric Engineering, Yanshan University, Qinhuangdao, China; ^2^College of Electronic Information Engineering, Hebei University, Baoding, China; ^3^Key Laboratory of Digital Medical Engineering of Hebei Province, Hebei University, Baoding, China; ^4^Development Planning Office, Affiliated Hospital of Hebei University, Baoding, China

**Keywords:** muscle fatigue, muscle network, functional corticomuscular coupling, information flow, EEG, sEMG

## Abstract

As a common neurophysiological phenomenon, voluntary muscle fatigue is accompanied by changes in both the central nervous system and peripheral muscles. Considering the effectiveness of the muscle network and the functional corticomuscular coupling (FCMC) in analyzing motor function, muscle fatigue can be analyzed by quantitating the intermuscular coupling and corticomuscular coupling. However, existing coherence-based research on muscle fatigue are limited by the inability of the coherence algorithm to identify the coupling direction, which cannot further reveal the underlying neural mechanism of muscle fatigue. To address this problem, we applied the time-delayed maximal information coefficient (TDMIC) method to quantitate the directional informational interaction in the muscle network and FCMC during a right-hand stabilized grip task. Eight healthy subjects were recruited to the present study. For the muscle networks, the beta-band information flow increased significantly due to muscle fatigue, and the information flow between the synergist muscles were stronger than that between the synergist and antagonist muscles. The information flow in the muscle network mainly flows to flexor digitorum superficialis (FDS), flexor carpi ulnar (FCU), and brachioradialis (BR). For the FCMC, muscle fatigue caused a significant decrease in the beta- and gamma-band bidirectional information flow. Further analysis revealed that the beta-band information flow was significantly stronger in the descending direction [electroencephalogram (EEG) to surface electromyography (sEMG)] than that in the ascending direction (sEMG to EEG) during pre-fatigue tasks. After muscle fatigue, the beta-band information flow in the ascending direction was significantly stronger than that in the descending direction. The present study demonstrates the influence of muscle fatigue on information flow in muscle networks and FCMC. We proposes that beta-band intermuscular and corticomuscular informational interaction plays an adjusting role in autonomous movement completion under muscle fatigue. Directed information flow analysis can be used as an effective method to explore the neural mechanism of muscle fatigue on the macroscopic scale.

## Introduction

Voluntary muscle fatigue is a common physiological phenomenon in daily life. It is usually defined as a decrease in the ability of the neuromuscular system to generate voluntary force during movement (Vollestad, 1997; Wang et al., 2017). Muscle fatigue research is of great significance in many fields, such as clinical analysis of neuromuscular diseases (Greig and Jones, 2010), sports medicine (Darvishani et al., 2019), and rehabilitation medicine (Arnall et al., 2002). With the development of non-invasive acquisition technology, the evaluation and prediction of muscle fatigue according to the characteristics of surface electromyography (sEMG) signals have been extensively studied (Bonato et al., 2001; Cifrek et al., 2009; Venugopal et al., 2014). However, it has been demonstrated that muscle fatigue is accompanied by changes in the central nervous system (Tanaka et al., 2011). Although changes in motor unit recruitment mediated by central mechanisms can be indirectly reflected in sEMG signals, it is difficult to analyze the neural mechanisms behind muscle fatigue at a systemic level by the single sEMG-based method.

Constructing muscle networks to analyze the synergistic characteristics between muscles has become a new method to explore the neuromuscular control mechanism recently (Boonstra et al., 2015; Kerkman et al., 2020; Houston et al., 2021). Muscle networks analysis quantifies the functional connectivity between motion-related muscles and is able to identify the frequency characteristics of specific muscles that are regulated by common neural inputs. Therefore, muscular networks analysis can reveal the characteristics of functional separation and integration for the neuromuscular system, which is suitable for analyzing the neuromuscular control mechanism behind muscle fatigue. On the other hand, it has been reported that the coupling relationship between scalp electroencephalogram (EEG) and sEMG in a specific frequency band can reflect the functional coupling between the motor cortex and effector muscles (Gwin and Ferris, 2012; Mehrkanoon et al., 2014). This coupling between EEG and sEMG was defined as functional corticomuscular coupling (FCMC). More and more studies verify the role of FCMC in different frequency bands during different force output tasks, providing important ideas to further reveal the underlying neural mechanism of muscle fatigue.

Recently, some researchers have used the coherence method to analyze the difference in FCMC during pre- and post-muscle fatigue (Yang et al., 2009; Siemionow et al., 2010; Tuncel et al., 2010; Wang et al., 2017). Studies based on muscle networks also mainly use coherence method to construct muscle networks (Kerkman et al., 2020; Houston et al., 2021). However, neurophysiological signals such as EEG and sEMG have been shown to be nonlinear and complex (Popivanov and Dushanova, 1999; Stam, 2005). The coherence method has some limitations in the comprehensive analysis of functional coupling. Additionally, autonomous movement is a result of the cerebral cortex driving muscle actions and the coupling between the motor cortex and effector muscles is directional (Mima et al., 2001; Witham et al., 2011). The information flow in the ascending (sEMG to EEG) and descending (EEG to sEMG) directions in FCMC has been shown to play a key role in neural information transmission for motor control and sensory feedback (Baker, 2007; Yang et al., 2018; Chen et al., 2019). As a normal neurophysiological phenomenon induced by voluntary movement, muscle fatigue inevitably leads to changes in the information interaction within the motor nervous system. Evaluation of the difference in information flow in different frequency bands for muscle networks and FCMC before and after muscle fatigue is helpful to better understand the functional mechanism of neural pathways underlying muscle fatigue. However, the coherence method cannot identify the direction of coupling, which limits its further application in this field.

The main aim of this work was to explore the motor regulation mechanism of the nervous system behind muscle fatigue by quantifying the information flow for muscle network and FCMC. Our previous research has demonstrated the effectiveness of the maximal information coefficient (MIC) and its improved algorithm (time-delayed maximal information coefficient, TDMIC) in FCMC research (Liang et al., 2020, 2021). TDMIC algorithm has been proved to be able to accurately identify the strength and direction of information flow between short-length nonlinear systems, so it is more suitable for the analysis of functional coupling between complex neurophysiological signals. The present study applied TDMIC to quantitatively analyze the changes of intermuscular- and corticomuscular-information flow with 30% steady-state grip during pre- and post-muscle fatigue tasks. To the best of our knowledge, this is the first study to analyze the directed information flow in muscle network and FCMC during pre- and post-muscle fatigue, providing a new perspective for understanding the neural mechanisms of muscle fatigue.

## Materials and Methods

### Subjects and Motor Task

Eight healthy right-handed subjects (seven males and one female; aged: 21–25 years) were recruited to the present study. All subjects were right-handed and assessed using the Edinburgh Inventory. All subjects gave informed consent prior to participation. The study was performed in accordance with the Declaration of Helsinki following approval by the Ethics Review Committee of the Affiliated Hospital of Hebei University (HDFY-LL-2020-091).

**FIGURE 1:**
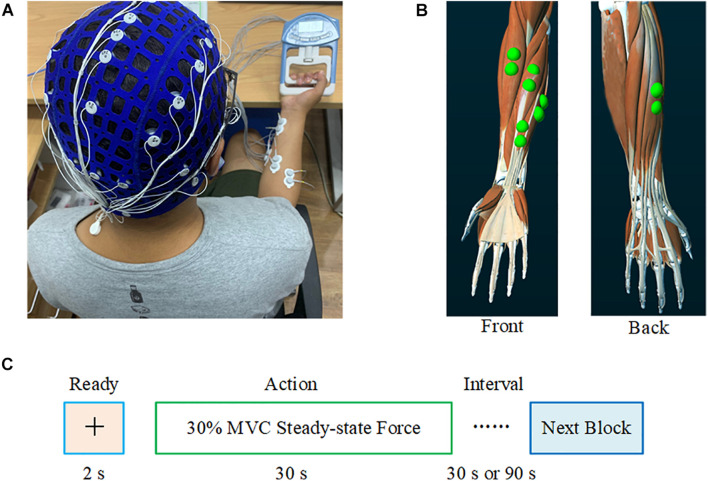
Experimental setting. **(A)** Synchronous recording of the subjects’ EEG and sEMG. **(B)** The electrode position on the FDS, FCU, FCR, BR, and ED. **(C)** The experimental paradigm. During the pre-fatigue task, the interval between each block was 90 s; during the post-fatigue task, this interval was 30 s.

Prior to the experiment, each subject performed three maximum voluntary contractions (MVCs) with a right-hand grip, and 30% of the average MVC value (Avg_MVC) before fatigue was taken as the target force for the experiment. Subjects used an electronic grip to perform the pre-fatigue task with 30% Avg_MVC of the right-hand grip for 30 s. Each subject repeated five blocks of the pre-fatigue task with 90-s breaks to avoid muscle fatigue. Each experiment included a 2-s preparation and a 30-s steady-state force output stage, as shown in Figure 1. Subsequently, the subjects performed the maximum grip strength continuously until ultimate endurance (i.e., muscle fatigue task). After this task, the subjects immediately performed a post-fatigue task; that is, maintaining 30% MVC of the right-hand grip for 30 s, similar to the pre-fatigue task. It should be noted that each subject repeated three blocks of the post-fatigue task with 30-s breaks to ensure completion of the experiment with muscle fatigue. After the experiment, the MVC values for the subjects were measured again to evaluate the impact of muscle fatigue on the ability to produce the maximum voluntary force.

### Data Recording and Preprocessing

Subjects sat comfortably on a chair, with the upper arm naturally placed vertically, the elbow angle maintained at 90°, and the right forearm placed on a support parallel to the ground. A 40-channel NeuroScan system (Neuroscan, Australia) was used to collect EEG and sEMG data simultaneously. Based on the international 10–20 system, the EEG signals from 32 scalp positions were recorded, with the binaural mastoid as a reference. EEG signals from the C3, C4, Cz, Pz, and Fz electrodes placed over the sensorimotor cortex were selected for subsequent analysis. To record the sEMG signal, bipolar Ag/AgCl electrodes (2 cm between the two poles) were attached to the skin above the flexor digitorum superficialis (FDS), flexor carpi ulnar (FCU), flexor carpi radialis (FCR), brachioradialis (BR), and extensor digitalis (ED) muscles of the right hand. Subjects washed their hair before the experiment and cleaned the skin with scrub where EMG electrodes were attached. The impedance of all electrodes was maintained below 5 KΩ. Subjects were advised to avoid blinking, swallowing, and turning their heads as much as possible during the experiment. EEG and sEMG were sampled at 1,024 Hz.

Only data from the steady-state force output stage of the experiment were included for further analysis. The 2–29 s data were selected for subsequent analysis to avoid the influence of large changes in grip strength at the start and end of the action. Each subject obtained four 28-s length epochs during pre- and post-fatigue for the right-hand grip. For EEG signals, 50 Hz power frequency interference was removed and a band-pass filtering (2–100 Hz) was performed. For sEMG signals, the data were band-pass filtered (5–500 Hz) and a notch filter was used to remove 50 Hz power frequency interference. After filtering, each epoch was cut into non-overlapping segments with a length of 1,000 ms. Then the EEG segments with obvious artifacts, such as blinking and neck rotation, were rejected by visual inspection. The corresponding sEMG segment was discarded. Next, independent component analysis (ICA) was used to remove artifacts such as electromyography and electrooculogram. ReMAE was used to further remove EMG artifacts from EEG signals (Chen et al., 2020). EEG and sEMG data in the beta (14–30 Hz) and gamma bands (31–45 Hz) were selected for further analysis. The EEG data recorded from C3 and the rectified sEMG data were used for further FCMC analysis.

### Data Analysis

#### Time-Delayed Maximal Information Coefficient

The MIC algorithm was proposed by Reshef et al. (2011) to measure the correlation between time series. MIC searches for the maximum mutual information by traversing all possible grid divisions of variables X and Y with a sample size *n* in the finite dataset *D* and regularizes it:

(1) $$\begin{array}{c} M\,I\,C\,\left(D\right)=\max_{x\,y<n^{0.6}}\left\{M\,{\left(D\right)}_{x,y}\right\}\\ =\max_{x\,y<n^{0.6}}\left\{\frac{\max(I\left(D,x,y\right)}{\log\min\left\{\text{x},y\right\}}\right\} \end{array}$$

where,_*I(D,x,y)*_ represents the mutual information of the two variables when the grid is divided into *x-by-y*. The computational workload can be reduced by setting *xy < n*^0.6^:

(2) $$I\,\left(D,\text{x},y\right)=\sum_{x\in X}\sum_{y\in Y}\text{p}\,\left(x,y\right)\,\log\left(\frac{p\,\left(x,y\right)}{p\,\left(x\right)\,p\,\left(y\right)}\right)$$

where, *p*(*x*, *y*) is the joint probability density function of *X* and *Y*, and *p*(*x*) and *p*(*y*) are their marginal probability density functions, respectively.

Since the MIC is symmetrical:

(3) MIC⁢(X,Y)=MIC⁢(Y,X)

As a result, the MIC algorithm cannot identify the coupling direction. To solve this problem, we introduced a time delay parameter and propose the following TDMIC algorithm (Liang et al., 2021):

(4) $$\begin{array}{c} T\,D\,M\,I\,C=M\,I\,C\,\left(X,Y,\tau \right)=\max_{x\,y<B\,\left(\text{n}\right)}\left\{\frac{\max(I_{G}\left(X,Y,\tau \right)}{\log\min\left\{\text{x},y\right\}}\right\}\\ =\max_{x\,y<B\,\left(\text{n}\right)}\left\{\frac{\max\left(\sum_{x_{t}}\sum_{y_{t-\tau }}\text{p}\,\left(x_{t},y_{t-\tau }\right)\,\log\left(\frac{p\,\left(x_{t},y_{t-\tau }\right)}{p\,\left(x_{t}\right)\,p\,\left(y_{t-\tau }\right)}\right)\right)}{\log\min\left\{\text{x},y\right\}}\right\} \end{array}$$

Similar to the time-delay mutual information method (TDMI), the time-delay symbol at which TDMIC reaches its peak was used to infer the direction of information flow between *X* and *Y* (Vastano and Swinney, 1988; Li et al., 2018).

To estimate the total information flow intensity over a period of time, the cumulated information flow within a certain delay *D* can be calculated using the following formula (Hinrichs et al., 2008)

(5) $$C_{T\,D\,M\,I\,C}=\sum_{i=1}^{D}T\,D\,M\,I\,C\,\left(k,i\right)$$

Here, delay *D* was set to 40 data points and *k* was set to 1.

#### Wavelet Transform

Considering that EEG and sEMG are both non-stationary and complex signals, wavelet transform with multi-resolution analysis characteristics can be used to capture more time-varying frequency information for EEG and sEMG during muscle fatigue (Karlsson et al., 1999, 2000; Liang et al., 2020). Therefore, the Morlet wavelet transform was used to obtain time-frequency energy maps for EEG and sEMG. represents the time series of channel *i* at time *t*, and the corresponding Morlet wavelet transform can be calculated as:

(6) $$W_{x_{i}}\,\left(t,f\right)\,\int x_{i}\,\left(\lambda \right)\,\phi_{t,f}^{*}\,\left(t-f\right)\,d\lambda$$

where, ϕ_*t*,*f*_^∗^ is the conjugate complex number for the mother wavelet function ϕ_*t*,*f*_^∗^; ϕ_*t*,*f*_(*λ*) = $A\cdot e^{i\,2\,\pi \,f\,\left(\lambda -t\right)}\cdot e^{\frac{-{\left(\lambda -t\right)}^{2}}{2\,\sigma^{2}}}$; *A* = ($\sigma \sqrt{2}){}^{-\frac{1}{2}}$ is the standardization factor, where $\sigma =\frac{8}{2\,\pi \,f}$.

**FIGURE 2:**
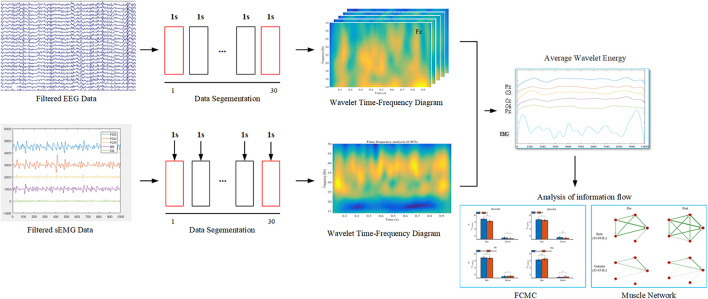
The procedure for EEG and sEMG data analysis. In the data segmentation phase, the 2–29 s data were selected for subsequent analysis.

Additionally, to quantitate the influence of muscle fatigue on EEG and sEMG wavelet energies, the cumulated wavelet energy in the beta (14–30 Hz) and gamma (31–45 Hz) bands were calculated separately. On the other hand, the beta- and gamma-band average wavelet energy of EEG and sEMG during pre- and post-muscle fatigue were used as the new time series for further muscle network and FCMC analysis. The procedure for EEG and sEMG data analysis as shown in Figure 2. Data were preprocessed and analyzed off-line in the MATLAB environment (R2018b, The MathWorks, Inc., Natick, MA, United States).

#### Statistical Analysis

In the present study, the permutation test was used to evaluate the significance of TDMIC (Schreiber and Schmitz, 2000). The specific implementation details are consistent with those of our previous research (Liang et al., 2020). To examine the effect of muscle fatigue on the cumulated wavelet energy and MIC, paired-sample *t*-tests were conducted. To compare the difference in cumulated information flow in different frequency bands and directions before and after muscle fatigue, a three-way repeated-measures analysis of variance (rANOVA) was performed with task (2 levels: pre-fatigue and post-fatigue), direction (2 levels: EEG to sEMG and sEMG to EEG), and frequency band (2 levels: beta and gamma) as the intra-subject factors, and the *C*_TDMIC_ value was the dependent variable. The significance level was set to 0.05 (α = 0.05). All statistical analyses were conducted in SPSS/PC version 20.0 (SPSS Inc., Chicago, IL, United States).

## Results

### Influences of Muscle Fatigue on EEG, sEMG, and MVC

All subjects completed the experimental tasks as required. Table 1 shows the MVC values during the pre- and post-fatigue tasks for all subjects. In comparison with the pre-fatigue task, we can see that the MVC for each subject was significantly decreased after the muscle fatigue task (average reduction of MVC: 12.3 ± 1.11 kg, *p* = 0.000).

**TABLE 1:** maximum voluntary contractions for all subjects.

Subject	Avg_MVC (kg)		30%MVC (kg)
		
	Pre	Post*	Pre
S1(M)	39.8	27.6	11.9
S2(M)	43.6	30.6	13.1
S3(M)	41	28.8	12.3
S4(M)	39.2	27.8	11.8
S5(M)	39.5	25.4	11.9
S6(M)	43	31	12.9
S7(M)	39.7	26.5	11.9
S8(F)	30	19.5	9.0

M denotes male; F denotes female. *p < 0.001.

**FIGURE 3:**
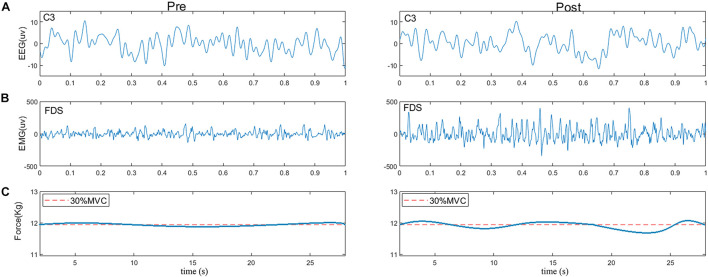
Typical examples of **(A)** raw EEG signals (C3), **(B)** raw sEMG signals (FDS), and **(C)** force performance during pre- and post-fatigue tasks. The red dotted line represents the target force.

Figure 3 shows typical examples of original EEG and sEMG signals during pre- and post-muscle fatigue. As observed, the amplitude of the sEMG signal was significantly increased due to muscle fatigue but the amplitude of the EEG signal did not change significantly. In addition, we recorded the tracking target force performance before and after muscle fatigue, as shown in Figure 3C. The ability of the subject to track the target force before fatigue was better than that after fatigue, with an obvious jitter in the grip curve after muscle fatigue.

**FIGURE 4:**
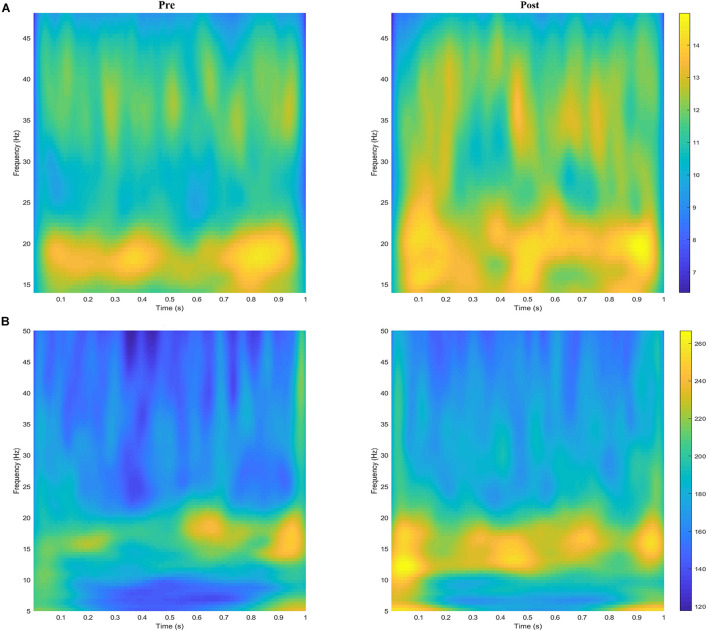
The grand averages of the wavelet time–frequency maps for **(A)** EEG (C3) and **(B)** sEMG (FDS) during the pre- and post-fatigue tasks.

The grand averages of the wavelet time–frequency maps for EEG and sEMG are shown in Figure 4. For EEG, the wavelet energy was mainly distributed in the frequency ranges of 16–21 and 31–45 Hz during the pre-fatigue task. During the post-fatigue task, the wavelet energy was mainly distributed in the frequency ranges of 14–30 and 30–45 Hz; the energy was stronger and over a wider range. For sEMG, the wavelet energy was mainly distributed in the 10–20 Hz frequency band during both the pre- and post-fatigue tasks, but the energy intensity was stronger after muscle fatigue.

**FIGURE 5:**
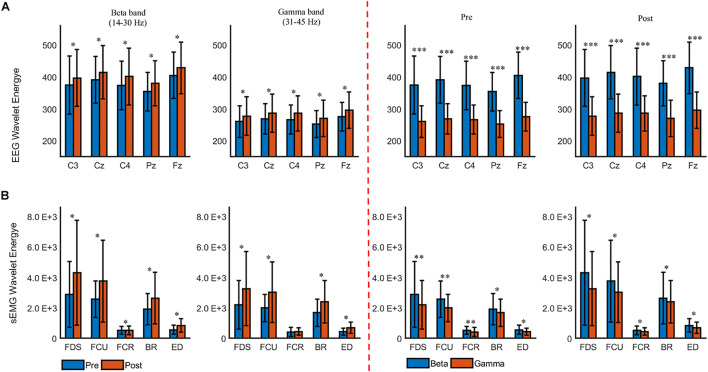
The grand averages of the beta- and gamma-band cumulated wavelet energy for **(A)** EEG and **(B)** sEMG during the pre- and post-fatigue tasks. ^∗^*p* < 0.05, ^∗∗^*p* < 0.01, ^∗∗∗^*p* < 0.001.

We further quantitated the difference in cumulated wavelet energy between the two muscle states. Figure 4 shows the grand average of the cumulated wavelet energy for EEG (Figure 5A) and sEMG (Figure 5B) in the beta and gamma bands during pre- and post-fatigue tasks. It can be seen from Figure 5A that at the five selected electrode positions (C3, Cz, C4, Pz, and Fz), the EEG wavelet energy after muscle fatigue was significantly higher than that before muscle fatigue (in the beta band, C3: *p* = 0.012, Cz: *p* = 0.013, C4: *p* = 0.027, Pz: *p* = 0.019, Fz: *p* = 0.015; in the gamma band, C3: *p* = 0.036, Cz: *p* = 0.017, C4: *p* = 0.030, Pz: *p* = 0.016, Fz: *p* = 0.016). The EEG wavelet energy in the beta band was significantly higher than that in the gamma band regardless of the state of the muscle (C3: *p* = 0.000, Cz: *p* = 0.000, C4: *p* = 0.000, Pz: *p* = 0.000, Fz: *p* = 0.000). Similarly, as shown in Figure 5B, the sEMG wavelet energy were also significantly increased due to muscle fatigue (in the beta band, FDS: *p* = 0.035, FCU: *p* = 0.031, FCR: *p* = 0.028, BR: *p* = 0.041, ED: *p* = 0.030; in the gamma band, FDS: *p* = 0.020, FCU: *p* = 0.035, FCR: *p* = 0.033, BR: *p* = 0.043, ED: *p* = 0.024). The sEMG wavelet energy in the beta band was significantly higher than that in the gamma band during both the pre- and post-fatigue tests (pre-fatigue: FDS: *p* = 0.003, FCU: *p* = 0.004, FCR: *p* = 0.006, BR: *p* = 0.023, ED: *p* = 0.043; post-fatigue: FDS: *p* = 0.032, FCU: *p* = 0.039, FCR: *p* = 0.028, BR: *p* = 0.022, ED: *p* = 0.030).

### The Influence of Muscle Fatigue on Muscle Network

**FIGURE 6:**
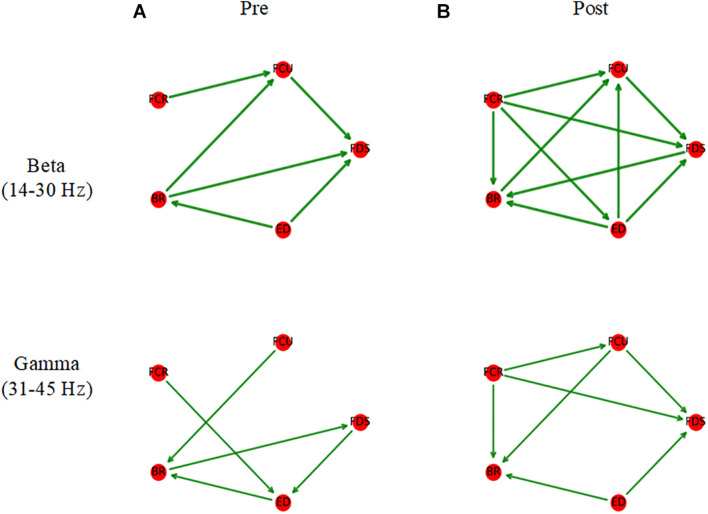
Average muscle networks pre- **(A)** and post-muscle fatigue **(B)**. The arrows indicate the stronger information flow direction, and the edge width is positively correlated with the strength of information flow. The average values of beta- and gamma-bands TDMIC before fatigue were used as display thresholds respectively to make network topology diagrams.

**FIGURE 7:**
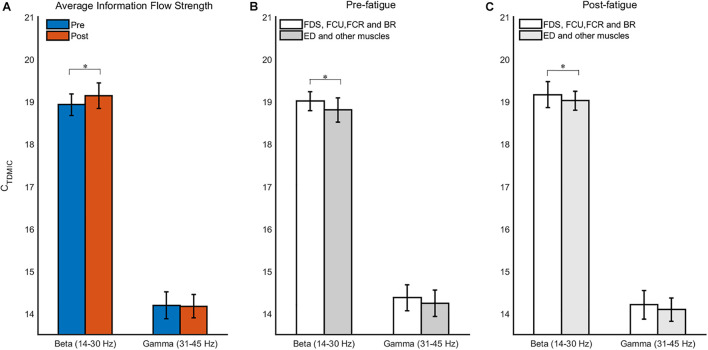
The grand averages of the information flow for muscle network. **(A)** Comparison of average information flow strength in beta- and gamma-bands before and after fatigue. **(B,C)** Comparison of information flow strength between different muscles before and after fatigue. ^∗^*p* < 0.05.

As shown in Figure 6, before muscle fatigue, information flow mainly exists among FDS, FCU, FCR, and BR, and mainly flows to FDS and FCU. Compared with pre-fatigue, Muscle network in the beta band was more tightly connected after fatigue and the Stronger information flow were mainly to FDS, FCU and BR. It’s worth noting that the connection between ED and other muscles were significantly increased after fatigue. As shown in Figure 7A, Compared with pre-fatigue, the beta-band intermuscular connectivity were significantly increased after muscle fatigue (*p* = 0.034). There was no significant difference in the strength of intramuscular coupling in gamma band before and after fatigue. It was also observed that the strength of intermuscular information flow in beta band was stronger than that in gamma band before and after fatigue (*p* = 0.000). In addition, as shown in Figures 7B,C, the beta-band information flow among FDS, FCU, FCR, and BR were significantly stronger than that between ED and these muscles (pre-fatigue: *p* = 0.040; post-fatigue: *p* = 0.035).

### The Influence of Muscle Fatigue on FCMC

**FIGURE 8:**
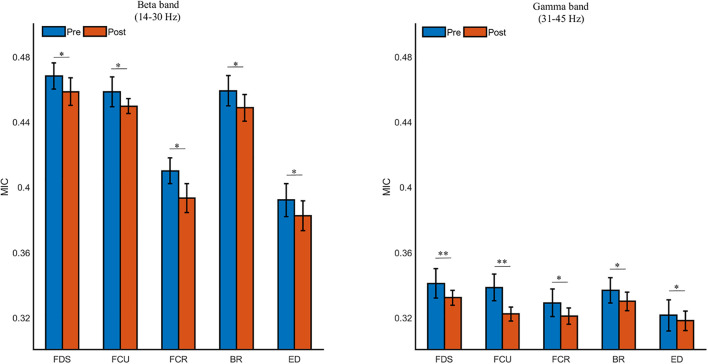
The grand averages of the beta- and gamma-band cortico-muscular connectivity during the pre- and post-fatigue tasks for FDS, FCU, FCR, BR, and ED. ^∗^*p* < 0.05, ^∗∗^*p* < 0.01.

As shown in Figure 8, the beta- and gamma-band MIC values to FDS was the strongest, followed by the FCU, BR, FCR, and ED (*p* < 0.05). The MIC values in the beta and gamma bands were also significantly decreased due to muscle fatigue (in the beta band, FDS: *p* = 0.018, FCU: *p* = 0.021, FCR: *p* = 0.033, BR: *p* = 0.016, ED: *p* = 0.040; in the gamma band, FDS: *p* = 0.006, FCU: *p* = 0.008, FCR: *p* = 0.023, BR: *p* = 0.031, ED: *p* = 0.025). Additionally, the MIC value in the beta band was significantly higher than that in the gamma band during both the pre- and post-fatigue tasks (*p* = 0.000).

**FIGURE 9:**
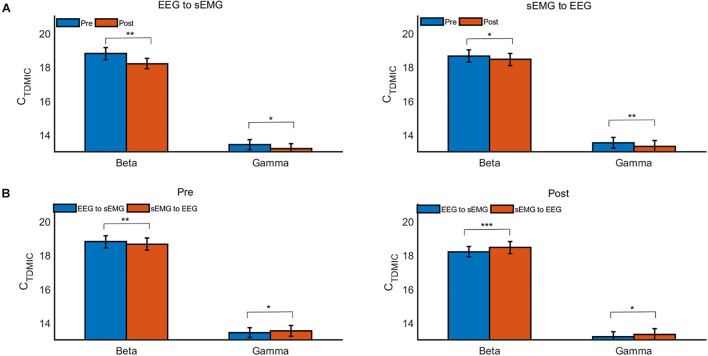
The grand average C_TDMIC_ values under different conditions. **(A)** Comparison of the C_TDMIC_ values between pre- and post-fatigue tasks under different conditions. **(B)** Comparison of the C_TDMIC_ values between the EEG to sEMG and the sEMG to EEG directions under different conditions. *p < 0.05, **p < 0.01.

Furthermore, we compared the cumulated bidirectional information flow in FCMC in the beta and gamma bands during the pre- and post-fatigue tasks, as shown in Figure 9. During the pre-fatigue task, the beta-band information flow in the descending direction (EEG to sEMG) was significantly higher than that in the ascending direction (sEMG to EEG) [*F*(1,7) = 20.33, *p* = 0.003, Bonferroni], while in the gamma band, the information flow in the ascending direction was significantly higher than that in the descending direction [*F*(1,7) = 6.33, *p* = 0.04, Bonferroni]. In comparison with the pre-fatigue task, the bidirectional information flow in the beta and gamma bands was significantly decreased during the post-fatigue task [beta band, ascending direction: *F*(1,7) = 7.94, *p* = 0.026, Bonferroni; beta band, descending direction: *F*(1,7) = 35.31, *p* = 0.001, Bonferroni; gamma band, ascending direction: *F*(1,7) = 13.24, *p* = 0.008, Bonferroni; gamma band, descending direction: *F*(1,7) = 7.41, *p* = 0.030, Bonferroni]. In particular, we observed that both the beta- and gamma-band information flow in the ascending direction were significantly higher than those in the descending direction during post-fatigue tasks [beta band: *F*(1,7) = 49.74, *p* = 0.000, Bonferroni; gamma band: *F*(1,7) = 5.93, *p* = 0.045, Bonferroni].

## Discussion

Muscle fatigue caused by voluntary movement is a common neurophysiological phenomenon. The purpose of the present research was to quantitatively analyze the influence of muscle fatigue on the information flow in muscle networks and FCMC using the improved MIC algorithm (TDMIC) and to explore the inner neural mechanism of muscle fatigue induced by voluntary movement. Our results show that muscle fatigue caused an increase in the strength of information flow in the muscle network and a decrease in the strength of information flow in the FCMC. In particular, for FCMC, we found that during the post-fatigue task, both the beta- and gamma-band information flow in the ascending direction were stronger than that in the descending direction, which was different from the stronger beta-than gamma-band information flow in the descending direction during the pre-fatigue task.

The EEG oscillation activity in the beta and gamma bands is related to the function of the sensorimotor cortex, and changes in the amplitude and spectral energy of the sEMG signal are also often used to assess muscle fatigue (Baker, 2007; van Duinen et al., 2007; Yang et al., 2009; Tuncel et al., 2010; Wang et al., 2017). Considering that even in the steady-state force output task, both the EEG and sEMG are time-variant, we used wavelet transform to analyze the energy changes of EEG and sEMG during the pre- and post-fatigue tasks. Benefiting from the trade-off in time–frequency resolution, wavelet transform has proven to be an effective method for analyzing non-stationary neural signals (Karlsson et al., 1999; Lu et al., 2011). It can be seen from the EEG wavelet time–frequency maps that the wavelet energy was mainly distributed in the beta and gamma bands. The EEG oscillating activity in these two frequency bands was generally considered to be related to the submaximal contractility task. Additionally, the fatigue task resulted in the diffusion of the EEG time–frequency energy band, which may be related to the non-linear adjustment of the cortex caused by muscle fatigue. Jing et al. used functional magnetic resonance imaging (FMRI) to demonstrate that the cortex experiences nonlinear modulation during muscle fatigue (Liu et al., 2002). Subsequent statistical analysis shows that muscle fatigue led to an increase in EEG and sEMG energy in the beta and gamma bands, which is consistent with previous reports (van Duinen et al., 2007; Yang et al., 2009; Berchicci et al., 2013; Wang et al., 2017). Since muscle fatigue results in decreased motor neuron activation in the motor cortex, increased energy in sensorimotor cortex regions (C3, C4, Cz, Fz, and Pz were selected in the present study) may indicate that more motor neurons in the central nervous system were recruited to process signals in order to maintain the established motor tasks and compensate for changes in the central and peripheral states. Similarly, for submaximal contraction, due to the decrease in force generation capacity during muscle fatigue, the increases in sEMG amplitude and energy were considered to reflect increased recruitment of additional motor units to maintain the strength level (Liu et al., 2003; Yang et al., 2009). Recently, Schlink et al. (2021) observed that fatigue induces altered EMG activation patterns in the medial gastrocnemius. They confirmed that this may be a protective mechanism of the neuromuscular system to avoid muscle injury by distributing the muscle load more broadly. This was also reflected in our sEMG wavelet time–frequency map.

The frequency characteristics of the coupling between sEMGs have been proved to reflect relevant information about the regulation of the neuromuscular system (Boonstra et al., 2015; Laine and Valero-Cuevas, 2017; Houston et al., 2021). In this study, consistent with previous studies, the information interaction of muscle network during motor task was mainly expressed in the beta and gamma bands, and the information flow in beta band was the strongest (Kattla and Lowery, 2010). This result may be related to the 30% MVC steady-state force output task paradigm. The intermuscular coupling in the beta band was proved to be correlated with the steady-state force output and could reflect the cooperative control strategy for the muscles under the common task (Kilner et al., 1999). Furthermore, we observed that muscle fatigue resulted in significantly increased information interaction and tighter connections between muscles in the beta band. This is consistent with previous studies based on hand fatigue tasks leading to increased intermuscular coupling (Danna-Dos Santos et al., 2010; Kattla and Lowery, 2010). The difference was that directed information flow analysis provided further directional information about the interactions between task-related muscles. Specifically, this study observed that due to muscle fatigue, the stronger information flow mainly flows to the synergistic muscles (i.e., FDS, FCU, and BR). Moreover, the information interaction between antagonistic muscles (i.e., ED) and other muscles also increases, which is mainly manifested as information flow from ED to other muscles. The increase in information interaction reflects the enhancement of coordination between task-related muscles, and to a certain extent reflects the adjustment of the control strategy for the neuromuscular system to maintain the level of the force output under the state of muscle fatigue. Additionally, this study observed that the information interaction between ED and other muscles (i.e., FDS, FCU, FCR, and BR) was weaker than that among these muscles. This difference in information interaction may be related to different muscle functions. In the process of grip task, ED is the antagonistic muscle for motor, while the other four muscles are the synergistic muscles. The observed differences in the strength of information interaction also provide evidence for the idea that antagonistic and synergistic muscles have different motor control mechanisms in the neuromuscular system. This is also consistent with previous researches (Kattla and Lowery, 2010).

Extensive studies have indicated that during the weak contraction task in limbs, the FCMC in the beta band is related to the control of steady-state force output (Conway et al., 1995; Mima et al., 2001), while that in the gamma band is related to the output of dynamic force and the integration of sensory information (Mima and Hallett, 1999; Omlor et al., 2007; Mehrkanoon et al., 2014; Liang et al., 2020). We observed significant beta- and gamma-band FCMC during sustained submaximal muscle contraction tasks, which is consistent with previous studies on the coupling between beta and gamma bands during submaximal muscle contraction (Mehrkanoon et al., 2014; Liang et al., 2020; Xie et al., 2020). In particular, the strongest cortico-muscular coupling was observed to the FDS muscle. One possible explanation for this phenomenon is that FDS plays an important role in the grip task. On the other hand, stronger cortico-muscular coupling in the distal muscles has also been demonstrated (Artoni et al., 2017). Further statistical analysis shows that the fatigue task caused a significant decrease in FCMC in the beta and gamma bands, which is consistent with the results of previous studies using the coherence method to analyze changes in corticomuscular coupling caused by muscle fatigue (Yang et al., 2009; Siemionow et al., 2010; Tuncel et al., 2010). FCMC reflects the informational interaction between the sensorimotor cortex and effector muscles, which was observed to be weakened by muscle fatigue in the present study. The weakening of FCMC can lead to a decrease in voluntary motor ability, which further led to a decrease in the subjects’ MVC and grip stability performance in the present study. The weakening of FCMC may be caused by a variety of mechanisms such as an inhibitory effect on the spinal motor neurons of the descending path or a decline in the information transmission function of the neuromuscular junction (Yang et al., 2009). Unfortunately, the possible mechanisms involve the directional specificity of information transmission in FCMC, which cannot be further effectively demonstrated by the coherence method since it lacks the ability to recognize coupling direction.

Increasing evidence indicates that FCMC may be simultaneously affected by both descending motor control commands and ascending sensory feedback information, thereby forming an oscillating sensory–motor loop (Mima et al., 2001; Witham et al., 2011; Campfens et al., 2013; Yang et al., 2018; Xie et al., 2020). Muscle fatigue caused by repetitive movements is a normal neurophysiological process which naturally involves changes in the transmission of motor control information in the nervous system. Unfortunately, previous studies on the effect of muscle fatigue on FCMC do not discuss this further. The results of statistical analysis performed in the present study show that before muscle fatigue, the strength of information flow in the beta band was significantly higher in the descending direction than that in the ascending direction, while this observation was the opposite in the gamma band. This may be related to the different roles of FCMC in different frequency bands for neural communication and interaction between the central nervous system and the effector muscles. The beta rhythm synchronous oscillation is generally considered to involve the transmission of descending motor control information and be responsible for the maintenance and output of steady-state force in submaximal contraction tasks (Baker, 2007; Lemon, 2008). In the experimental paradigm of the present study, the stability of the grip was better during the pre-fatigue task, and the transmission of the descending control information was dominant during the continuous steady-state force output in the beta band. The beta-band results are consistent with those of previous studies on steady-state force output tasks (Mima et al., 2001; Chen et al., 2019). The synchronized oscillation of the gamma rhythm is considered to be related to the generation of dynamic forces and the integration of information such as attention, vision, and proprioception (Omlor et al., 2007; Mehrkanoon et al., 2014), which may also explain our observation that the ascending information flow in the gamma band was dominant.

Following the occurrence of muscle fatigue, the strength of the bidirectional information flow in the beta and gamma bands decreased significantly, which is consistent with the decrease in FCMC caused by muscle fatigue. The decrease in the descending information flow also provides partial evidence for the above-mentioned muscle fatigue leading to inhibitory effects on the descending path. Previous studies on the motor cortex using transcranial magnetic stimulation have also confirmed that muscle fatigue leads to a decrease in excitatory input from the motor cortex to the effector muscles (Taylor and Gandevia, 2001; Sogaard et al., 2006). Another study also confirmed the influence of muscle fatigue on proprioception (Voight et al., 1996). The observed decrease in the ascending information flow may reflect a decline in the transmission function of sensory feedback information, thus affecting the subjects’ proprioception. In particular, unlike during the pre-fatigue task, we found that during the post-fatigue task, both the beta- and gamma-band information flow in the ascending direction were higher than those in the descending direction. This change may be related to the active adaptation of the sensory motor nervous system to muscle fatigue. As muscle fatigue progressed, the information transmission of the descending motor pathway for the subjects was inhibited and the production of voluntary force was reduced; however, relatively more sensory feedback information needed to be integrated to dynamically adapt to the decrease in grip stability. Chen et al. (2019) previously reported that the beta rhythm oscillations can regulate the information transmission between the sensorimotor cortex and effector muscles. Therefore, it is reasonable to suggest that in order to complete the target force task, the beta-band information flow between the cortex and effector muscle may be dynamically adjusted as the task environment changes (e.g., caused by muscle fatigue in the present study). In essence, the changes in information interaction are also a guarantee that the subjects complete autonomous movements in the state of muscle fatigue.

## Conclusion

This research focused on the influence of muscle fatigue on information flow in muscle networks and FCMC. In this study, we found different connection patterns of synergistic and antagonistic muscles in the muscle network. Furthermore, muscle fatigue resulted in an increase in information flow in the muscle network and a significant decrease in the bidirectional information flow in the FCMC. Additionally, bidirectional information flow in FCMC showed different frequency specificity before and after muscle fatigue. We demonstrate that quantitating the information flow in muscle network and FCMC can help to explore the neural mechanisms of muscle fatigue. At the same time, we confirm that the dynamic adjustment of the beta-band information flow in the muscle network and in the FCMC is very important for maintaining the stable force output level, especially under the condition of muscle fatigue.

## Data Availability Statement

The raw data supporting the conclusions of this article will be made available by the authors, without undue reservation.

## Ethics Statement

The studies involving human participants were reviewed and approved by Ethics Review Committee of the Affiliated Hospital of Hebei University (HDFY-LL-2020-091). The patients/participants provided their written informed consent to participate in this study.

## Author Contributions

TL involved in development, computational model, performed the experiment design, data collection, analysis, and writing of the manuscript. QZ and LH contributed to the experiment design, data collection, and analysis. XL and BD assisted with the data collection and analysis. XL and HW contributed to the funding, experiment design, provision of resources, and writing. All authors read and approved the final version of the manuscript.

## Conflict of Interest

The authors declare that the research was conducted in the absence of any commercial or financial relationships that could be construed as a potential conflict of interest.

## Publisher’s Note

All claims expressed in this article are solely those of the authors and do not necessarily represent those of their affiliated organizations, or those of the publisher, the editors and the reviewers. Any product that may be evaluated in this article, or claim that may be made by its manufacturer, is not guaranteed or endorsed by the publisher.
